# Ethyl 5-amino-1-(6-chloro­pyridazin-3-yl)-1*H*-pyrazole-4-carboxyl­ate

**DOI:** 10.1107/S1600536810034240

**Published:** 2010-08-28

**Authors:** Abdul Qayyum Ather, M. Nawaz Tahir, Misbahul Ain Khan, Muhammad Makshoof Athar, Eliana Aparecida Silicz Bueno

**Affiliations:** a Department of Chemistry, Islamia University, Bahawalpur, Pakistan; b Applied Chemistry Research Center, PCSIR Laboratories Complex, Lahore 54600, Pakistan; c Department of Physics, University of Sargodha, Sargodha, Pakistan; d Institute of Chemistry, University of the Punjab, Lahore, Pakistan; e Instituto de Quimica, Universidade Estadual de Londrina, Londrina, Pr., Brazil

## Abstract

In the title compound, C_10_H_10_ClN_5_O_2_, the dihedral angle between the aromatic rings is 0.16 (9)°. Two *S*(6) ring motifs are formed due to intra­molecular N—H⋯N and N—H⋯O hydrogen bonds. In the crystal, inversion dimers linked by pairs of N—H⋯N hydrogen bonds generate *R*_2_^2^(14) [or *R*_4_^4^(10) *via* the intra­molecular hydrogen bonds] ring motifs. Polymeric chains propagating in [210] are formed as a result of inter­linking the dimers by pairs of C—H⋯N inter­actions, completing *R*_2_^2^(6) ring motifs.

## Related literature

For biochemical background and related structures, see: Ather *et al.* (2010**a* ▶,*b* ▶,c* ▶). For graph-set notation, see: Bernstein *et al.* (1995 ▶).
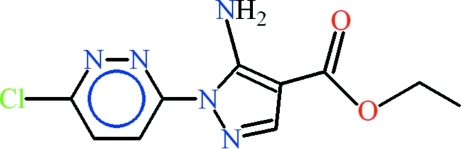


## Experimental

### 

#### Crystal data

C_10_H_10_ClN_5_O_2_
*M* *_r_* = 267.68
Triclinic, 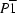

*a* = 5.3618 (3) Å
*b* = 8.6168 (4) Å
*c* = 13.1585 (7) Å
α = 77.734 (2)°
β = 82.928 (1)°
γ = 86.722 (2)°
*V* = 589.24 (5) Å^3^
*Z* = 2
Mo *K*α radiation
μ = 0.33 mm^−1^
*T* = 296 K
0.25 × 0.20 × 0.08 mm

#### Data collection

Bruker Kappa APEXII CCD diffractometer
Absorption correction: multi-scan (*SADABS*; Bruker, 2005 ▶) *T*_min_ = 0.982, *T*_max_ = 0.988
8832 measured reflections
2125 independent reflections
1721 reflections with *I* > 2σ(*I*)
*R*_int_ = 0.032

#### Refinement

*R*[*F* ^2^ > 2σ(*F* ^2^)] = 0.035
*wR*(*F* ^2^) = 0.101
*S* = 1.06
2125 reflections
164 parameters
H-atom parameters constrained
Δρ_max_ = 0.22 e Å^−3^
Δρ_min_ = −0.16 e Å^−3^

### 

Data collection: *APEX2* (Bruker, 2009 ▶); cell refinement: *SAINT* (Bruker, 2009 ▶); data reduction: *SAINT*; program(s) used to solve structure: *SHELXS97* (Sheldrick, 2008 ▶); program(s) used to refine structure: *SHELXL97* (Sheldrick, 2008 ▶); molecular graphics: *ORTEP-3* (Farrugia, 1997 ▶) and *PLATON* (Spek, 2009 ▶); software used to prepare material for publication: *WinGX* (Farrugia, 1999 ▶) and *PLATON*.

## Supplementary Material

Crystal structure: contains datablocks global, I. DOI: 10.1107/S1600536810034240/hb5619sup1.cif

Structure factors: contains datablocks I. DOI: 10.1107/S1600536810034240/hb5619Isup2.hkl

Additional supplementary materials:  crystallographic information; 3D view; checkCIF report

## Figures and Tables

**Table 1:** Hydrogen-bond geometry (Å, °)

*D*—H⋯*A*	*D*—H	H⋯*A*	*D*⋯*A*	*D*—H⋯*A*
N5—H5*A*⋯N1	0.86	2.17	2.775 (2)	127
N5—H5*B*⋯O2	0.86	2.40	2.942 (2)	122
N5—H5*B*⋯N2^i^	0.86	2.41	3.017 (2)	128
C5—H5⋯N4^ii^	0.93	2.53	3.313 (2)	142

Symmetry codes: (i) 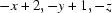; (ii) 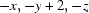.

**Fig. 1.**
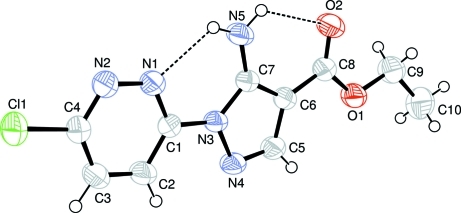
View of (I) with displacement ellipsoids drawn at the 50% probability level. H-atoms are shown as small spheres of arbitrary radius. The dotted lines indicate the intramolecular H-bonds.

**Fig. 2.**
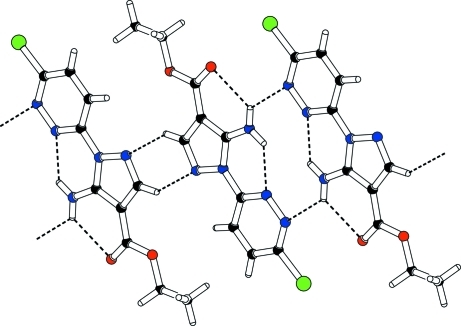
Packing diagram of (I) showing that the molecules form dimers, which are interlinked in the form of polymeric chains.

## supplementary crystallographic information

### Comment

In continuation of our studies of pyrazolylpyridazine derivatives (Ather *et al.*, 2010**a*, b, c*), the title compound (I, Fig. 1) is being reported here.

In (I), the 1-(6-chloropyridazin-3-yl)-1*H*-pyrazol-5-amine moiety A (C1—C7/N1—N5/CL1) and ethyl formate group B (C8—C10/O1/O2) are planar with r. m. s. deviations of 0.0026 and 0.0293 Å, respectively. The dihedral angle between A/B is 3.09 (12)°. There exist two S(6) ring motifs (Bernstein *et al.*, 1995) due to N–H···N and N—H···O types of intramolecular H-bondings (Table 1, Fig. 1). The molecules are dimerized due to N–H···N type of H-bonding (Table 2, Fig. 2) with *R*_4_^4^(10) ring motifs. The dimers are interliked in the from of polymeric chains due to H-bondings of C—H···N type with *R*_2_^2^(6) ring motifs (Table 2, Fig. 2).

### Experimental

3-Chloro-6-hydrazinylpyridazine (2 g, 13.84 mmol) and ethylethoxymethylene cyanoacetate (2.35 g, 13.84 mmol) were dissolved in acetic acid (10 ml). The obtained reaction mixture was refluxed for 4 h and cooled to room temperature. The resulting product was poured in 100 ml of distiled water and the precipitates were formed. The precipitates obtained by filteration were washed three times by water. The crude material obtained was dried and purified by column chromatography. The final product was re-crystallized in benzene to obtain light brown plates of (I).

### Refinement

The H-atoms were positioned geometrically (N–H = 0.86, C–H = 0.93–0.97 Å) and were included in the refinement in the riding model approximation, with *U*_iso_(H) = x*U*_eq_(C, N), where x = 1.5 for methyl and x = 1.2 for all other H-atoms.

### Crystal data

**Table:**

C_10_H_10_ClN_5_O_2_	*Z* = 2
*M**_r_* = 267.68	*F*(000) = 276
Triclinic, *P*1	*D*_x_ = 1.509 Mg m^−^^3^
Hall symbol: -P 1	Mo *K*α radiation, λ = 0.71073 Å
*a* = 5.3618 (3) Å	Cell parameters from 1721 reflections
*b* = 8.6168 (4) Å	θ = 2.4–25.2°
*c* = 13.1585 (7) Å	µ = 0.33 mm^−^^1^
α = 77.734 (2)°	*T* = 296 K
β = 82.928 (1)°	Plate, light brown
γ = 86.722 (2)°	0.25 × 0.20 × 0.08 mm
*V* = 589.24 (5) Å^3^	

### Data collection

**Table:**

Bruker Kappa APEXII CCD diffractometer	2125 independent reflections
Radiation source: fine-focus sealed tube	1721 reflections with *I* > 2σ(*I*)
graphite	*R*_int_ = 0.032
Detector resolution: 8.10 pixels mm^-1^	θ_max_ = 25.2°, θ_min_ = 2.4°
ω scans	*h* = −6→6
Absorption correction: multi-scan (*SADABS*; Bruker, 2005)	*k* = −10→10
*T*_min_ = 0.982, *T*_max_ = 0.988	*l* = −15→15
8832 measured reflections	

### Refinement

**Table:**

Refinement on *F*^2^	Primary atom site location: structure-invariant direct methods
Least-squares matrix: full	Secondary atom site location: difference Fourier map
*R*[*F*^2^ > 2σ(*F*^2^)] = 0.035	Hydrogen site location: inferred from neighbouring sites
*wR*(*F*^2^) = 0.101	H-atom parameters constrained
*S* = 1.06	*w* = 1/[σ^2^(*F*_o_^2^) + (0.049*P*)^2^ + 0.1261*P*] where *P* = (*F*_o_^2^ + 2*F*_c_^2^)/3
2125 reflections	(Δ/σ)_max_ = 0.001
164 parameters	Δρ_max_ = 0.22 e Å^−^^3^
0 restraints	Δρ_min_ = −0.16 e Å^−^^3^

### Special details

**Table:**

Geometry. Bond distances, angles *etc*. have been calculated using the rounded fractional coordinates. All su's are estimated from the variances of the (full) variance-covariance matrix. The cell e.s.d.'s are taken into account in the estimation of distances, angles and torsion angles
Refinement. Refinement of *F*^2^ against ALL reflections. The weighted *R*-factor *wR* and goodness of fit *S* are based on *F*^2^, conventional *R*-factors *R* are based on *F*, with *F* set to zero for negative *F*^2^. The threshold expression of *F*^2^ > σ(*F*^2^) is used only for calculating *R*-factors(gt) *etc*. and is not relevant to the choice of reflections for refinement. *R*-factors based on *F*^2^ are statistically about twice as large as those based on *F*, and *R*- factors based on ALL data will be even larger.

### Fractional atomic coordinates and isotropic or equivalent isotropic displacement parameters (Å^2^)

**Table:**

	*x*	*y*	*z*	*U*_iso_*/*U*_eq_	
Cl1	1.21255 (10)	0.81971 (7)	−0.39777 (4)	0.0677 (2)	
O1	0.0151 (3)	0.69934 (15)	0.27701 (10)	0.0604 (4)	
O2	0.3413 (3)	0.52161 (16)	0.27988 (10)	0.0640 (5)	
N1	0.8615 (3)	0.69752 (18)	−0.12132 (12)	0.0510 (5)	
N2	1.0286 (3)	0.70752 (18)	−0.20741 (12)	0.0542 (5)	
N3	0.4983 (2)	0.78156 (16)	−0.03130 (10)	0.0431 (5)	
N4	0.2872 (3)	0.88448 (17)	−0.03037 (12)	0.0502 (5)	
N5	0.6846 (3)	0.56546 (17)	0.08430 (12)	0.0555 (5)	
C1	0.6639 (3)	0.79680 (19)	−0.12305 (13)	0.0415 (5)	
C2	0.6174 (3)	0.9141 (2)	−0.21063 (15)	0.0533 (6)	
C3	0.7854 (3)	0.9223 (2)	−0.29680 (15)	0.0561 (6)	
C4	0.9885 (3)	0.8155 (2)	−0.29040 (14)	0.0481 (6)	
C5	0.1714 (3)	0.8415 (2)	0.06357 (14)	0.0500 (6)	
C6	0.2931 (3)	0.7149 (2)	0.12671 (13)	0.0445 (5)	
C7	0.5052 (3)	0.67718 (19)	0.06360 (13)	0.0422 (5)	
C8	0.2250 (3)	0.6341 (2)	0.23350 (14)	0.0486 (6)	
C9	−0.0707 (5)	0.6236 (3)	0.38284 (17)	0.0732 (8)	
C10	−0.2913 (4)	0.7155 (3)	0.42191 (19)	0.0806 (9)	
H2	0.47768	0.98310	−0.20953	0.0639*	
H3	0.76539	0.99675	−0.35797	0.0673*	
H5	0.02210	0.89012	0.08673	0.0600*	
H5A	0.80622	0.55512	0.03686	0.0665*	
H5B	0.67887	0.50366	0.14512	0.0665*	
H9A	0.06306	0.61935	0.42685	0.0879*	
H9B	−0.11731	0.51568	0.38514	0.0879*	
H10A	−0.42248	0.71987	0.37778	0.1208*	
H10B	−0.24295	0.82139	0.42099	0.1208*	
H10C	−0.35108	0.66460	0.49222	0.1208*	

### Atomic displacement parameters (Å^2^)

**Table:**

	*U*^11^	*U*^22^	*U*^33^	*U*^12^	*U*^13^	*U*^23^
Cl1	0.0694 (3)	0.0765 (4)	0.0499 (3)	0.0052 (3)	0.0126 (2)	−0.0098 (3)
O1	0.0675 (8)	0.0591 (8)	0.0434 (7)	0.0132 (6)	0.0109 (6)	0.0005 (6)
O2	0.0691 (9)	0.0651 (9)	0.0483 (8)	0.0172 (7)	−0.0037 (7)	0.0029 (6)
N1	0.0510 (8)	0.0536 (9)	0.0424 (8)	0.0121 (7)	0.0004 (7)	−0.0037 (7)
N2	0.0530 (9)	0.0583 (10)	0.0457 (9)	0.0122 (7)	0.0024 (7)	−0.0064 (8)
N3	0.0442 (8)	0.0407 (8)	0.0398 (8)	0.0096 (6)	−0.0023 (6)	−0.0027 (6)
N4	0.0469 (8)	0.0485 (8)	0.0486 (9)	0.0168 (7)	−0.0014 (7)	−0.0022 (7)
N5	0.0544 (9)	0.0579 (10)	0.0445 (9)	0.0196 (7)	−0.0021 (7)	0.0032 (7)
C1	0.0422 (9)	0.0420 (9)	0.0395 (9)	0.0041 (7)	−0.0040 (7)	−0.0085 (7)
C2	0.0535 (10)	0.0515 (10)	0.0480 (11)	0.0137 (8)	−0.0039 (8)	−0.0003 (8)
C3	0.0608 (11)	0.0570 (11)	0.0428 (10)	0.0081 (9)	−0.0026 (9)	0.0026 (9)
C4	0.0512 (10)	0.0512 (10)	0.0403 (10)	0.0011 (8)	−0.0008 (8)	−0.0093 (8)
C5	0.0472 (9)	0.0500 (10)	0.0479 (10)	0.0106 (8)	0.0013 (8)	−0.0064 (8)
C6	0.0462 (9)	0.0451 (9)	0.0390 (9)	0.0058 (7)	−0.0027 (7)	−0.0050 (8)
C7	0.0441 (9)	0.0404 (9)	0.0400 (9)	0.0047 (7)	−0.0058 (7)	−0.0050 (7)
C8	0.0526 (10)	0.0482 (10)	0.0429 (10)	0.0042 (8)	−0.0034 (8)	−0.0073 (8)
C9	0.0894 (15)	0.0670 (13)	0.0492 (12)	0.0089 (11)	0.0189 (11)	0.0010 (10)
C10	0.0777 (15)	0.0906 (17)	0.0658 (15)	0.0008 (13)	0.0197 (12)	−0.0161 (13)

### Geometric parameters (Å, °)

**Table:**

Cl1—C4	1.7337 (18)	C2—C3	1.351 (3)
O1—C8	1.348 (2)	C3—C4	1.384 (2)
O1—C9	1.438 (3)	C5—C6	1.405 (2)
O2—C8	1.214 (2)	C6—C7	1.389 (2)
N1—N2	1.346 (2)	C6—C8	1.442 (2)
N1—C1	1.323 (2)	C9—C10	1.486 (4)
N2—C4	1.307 (2)	C2—H2	0.9300
N3—N4	1.398 (2)	C3—H3	0.9300
N3—C1	1.395 (2)	C5—H5	0.9300
N3—C7	1.378 (2)	C9—H9A	0.9700
N4—C5	1.301 (2)	C9—H9B	0.9700
N5—C7	1.332 (2)	C10—H10A	0.9600
N5—H5A	0.8600	C10—H10B	0.9600
N5—H5B	0.8600	C10—H10C	0.9600
C1—C2	1.400 (2)		
			
C8—O1—C9	115.59 (16)	N3—C7—N5	124.20 (15)
N2—N1—C1	119.35 (15)	N3—C7—C6	105.71 (14)
N1—N2—C4	118.42 (16)	O1—C8—O2	123.39 (16)
N4—N3—C1	118.30 (13)	O1—C8—C6	111.74 (15)
N4—N3—C7	111.47 (12)	O2—C8—C6	124.87 (16)
C1—N3—C7	130.23 (13)	O1—C9—C10	109.1 (2)
N3—N4—C5	104.02 (14)	C1—C2—H2	122.00
H5A—N5—H5B	120.00	C3—C2—H2	122.00
C7—N5—H5A	120.00	C2—C3—H3	121.00
C7—N5—H5B	120.00	C4—C3—H3	121.00
N1—C1—N3	116.76 (15)	N4—C5—H5	123.00
N1—C1—C2	123.38 (16)	C6—C5—H5	123.00
N3—C1—C2	119.86 (15)	O1—C9—H9A	110.00
C1—C2—C3	116.75 (16)	O1—C9—H9B	110.00
C2—C3—C4	117.32 (17)	C10—C9—H9A	110.00
N2—C4—C3	124.78 (17)	C10—C9—H9B	110.00
Cl1—C4—C3	119.87 (14)	H9A—C9—H9B	108.00
Cl1—C4—N2	115.35 (13)	C9—C10—H10A	109.00
N4—C5—C6	113.70 (15)	C9—C10—H10B	109.00
C5—C6—C7	105.11 (15)	C9—C10—H10C	109.00
C5—C6—C8	130.39 (16)	H10A—C10—H10B	109.00
C7—C6—C8	124.51 (15)	H10A—C10—H10C	109.00
N5—C7—C6	130.09 (16)	H10B—C10—H10C	109.00
			
C9—O1—C8—O2	−2.0 (3)	C1—N3—C7—C6	−179.82 (16)
C9—O1—C8—C6	178.45 (17)	N3—N4—C5—C6	0.2 (2)
C8—O1—C9—C10	176.03 (17)	N1—C1—C2—C3	0.6 (3)
C1—N1—N2—C4	−0.3 (2)	N3—C1—C2—C3	179.95 (16)
N2—N1—C1—N3	−179.52 (15)	C1—C2—C3—C4	−0.6 (2)
N2—N1—C1—C2	−0.1 (3)	C2—C3—C4—Cl1	−179.69 (14)
N1—N2—C4—Cl1	−179.85 (13)	C2—C3—C4—N2	0.2 (3)
N1—N2—C4—C3	0.2 (3)	N4—C5—C6—C7	−0.2 (2)
C1—N3—N4—C5	179.72 (14)	N4—C5—C6—C8	−179.72 (17)
C7—N3—N4—C5	0.00 (18)	C5—C6—C7—N3	0.21 (18)
N4—N3—C1—N1	−179.93 (15)	C5—C6—C7—N5	−179.42 (18)
N4—N3—C1—C2	0.6 (2)	C8—C6—C7—N3	179.73 (16)
C7—N3—C1—N1	−0.3 (3)	C8—C6—C7—N5	0.1 (3)
C7—N3—C1—C2	−179.69 (16)	C5—C6—C8—O1	−2.9 (3)
N4—N3—C7—N5	179.53 (15)	C5—C6—C8—O2	177.57 (18)
N4—N3—C7—C6	−0.13 (18)	C7—C6—C8—O1	177.73 (16)
C1—N3—C7—N5	−0.2 (3)	C7—C6—C8—O2	−1.8 (3)

### Hydrogen-bond geometry (Å, °)

**Table:**

*D*—H···*A*	*D*—H	H···*A*	*D*···*A*	*D*—H···*A*
N5—H5A···N1	0.86	2.17	2.775 (2)	127
N5—H5B···O2	0.86	2.40	2.942 (2)	122
N5—H5B···N2^i^	0.86	2.41	3.017 (2)	128
C5—H5···N4^ii^	0.93	2.53	3.313 (2)	142

Symmetry codes: (i) −*x*+2, −*y*+1, −*z*; (ii) −*x*, −*y*+2, −*z*.
